# Gestational Trophoblastic Disease in a Resource-Limited Setting: A Prospective Study of Molar Pregnancy at a Teaching Hospital in Northeast India

**DOI:** 10.7759/cureus.109770

**Published:** 2026-05-27

**Authors:** Sushamanka Das, Goter Doke, Amrita Datta

**Affiliations:** 1 Obstetrics and Gynecology, All India Institute of Medical Sciences Guwahati, Guwahati, IND; 2 Obstetrics and Gynecology, Tomo Riba Institute Health and Medical Sciences (TRIHMS), Naharlagun, IND

**Keywords:** gestational trophoblastic disease, human chorionic gonadotropin, hydatidiform mole, prospective studies, tertiary care teaching hospital

## Abstract

Background

Molar pregnancy is a premalignant form of gestational trophoblastic disease with a higher incidence reported in Asian populations. Data from Northeast India, where geographic and healthcare disparities may influence disease patterns and outcomes, remain limited.

Methods

This prospective observational study was conducted over 15 months at a tertiary care teaching hospital in Northeast India. Women aged 15-49 years with ultrasonography- and histopathology-confirmed molar pregnancy were included. Clinical and treatment data were recorded, and patients were followed with serial serum β-human chorionic gonadotropin (β-hCG) until normalization. Predictors of chemotherapy requirement were analyzed using logistic regression and ROC curve analysis.

Results

The mean age was 27.8±5.5 years. Common presenting features included amenorrhoea and vaginal bleeding. Complete mole was the predominant subtype (75.9%). Suction evacuation was performed in 83.3% of patients as primary treatment, while 22.2% needed chemotherapy. On multivariate analysis, β-hCG ≥100,000 mIU/mL (aOR 4.9, p=0.003) and uterine size greater than gestational age (aOR 2.8, p=0.048) were independent predictors of chemotherapy. Receiver operating characteristic (ROC) analysis showed good predictive performance (AUC 0.78). Ultrasonography showed moderate agreement with histopathology (kappa=0.62).

Discussion

The age and socioeconomic patterns differed from traditional risk profiles, suggesting regional and genetic influences on disease distribution. Management outcomes are favourable, supported by improved imaging facilities, effective treatment, and meticulous surveillance. β-hCG demonstrates good discriminatory ability and may aid early risk stratification. Larger multicentric studies are needed in the future.

## Introduction

Molar pregnancy, or hydatidiform mole, is a premalignant form of gestational trophoblastic disease (GTD) characterised by abnormal proliferation of trophoblastic tissue and hydropic degeneration of chorionic villi [1]. It results from aberrant fertilization and is broadly classified into complete and partial mole based on distinct genetic, morphological, and clinical features. While complete moles are typically androgenetic and lack fetal tissue, partial moles are usually triploid and may be associated with an abnormal fetus [2].

The incidence of molar pregnancy varies widely across geographical regions, with significantly higher rates reported in Asia as compared with Western countries. Estimates suggest an incidence of approximately 1 in 400 pregnancies in India, compared with 1 in 1000-2000 pregnancies in Europe and North America [3]. These variations have been attributed to differences in nutritional status, socioeconomic conditions, reproductive patterns, and access to healthcare services. Established risk factors include extremes of maternal age, prior molar pregnancy, high parity, and possible micronutrient deficiencies, particularly vitamin A and folate [4-6].

Clinically, molar pregnancy most commonly presents with first-trimester vaginal bleeding, often accompanied by excessive nausea and vomiting, uterine enlargement disproportionate to gestational age, and markedly elevated serum β-human chorionic gonadotropin (β-hCG) levels [6,7]. Ultrasonography plays a pivotal role in early diagnosis, with the characteristic “snowstorm” appearance in a complete mole, facilitating prompt identification. Early detection is critical, as untreated cases may progress to gestational trophoblastic neoplasia (GTN), a potentially malignant but highly curable condition with timely intervention [6]. Management primarily involves evacuation of uterine contents, followed by serial β-hCG monitoring to ensure complete remission and to detect persistent disease. The majority of cases respond well to this approach; however, a subset requires chemotherapy, particularly in cases progressing to GTN. Early diagnosis and chemotherapy using methotrexate or dactinomycin yield excellent outcomes in high-risk cases [8].

Despite its clinical significance, there is a paucity of data from northeastern parts of India, where healthcare access and referral facilities, as well as the unique sociocultural settings, may influence the disease presentation and outcomes. Most available studies limit the generalisability of findings to this geographically distinct population.

Hence, this study was designed with dual objectives: first, to establish the epidemiological and clinical profile of molar pregnancy at a tertiary center in Northeast India; and second, to evaluate post-evacuation clinical outcomes and identify independent risk factors predicting the requirement for subsequent chemotherapy.

## Materials and methods

Methodology

This single-center, prospective observational study was conducted in the Department of Obstetrics and Gynecology at a tertiary care teaching hospital in Arunachal Pradesh in Northeast India over a period of 15 months (November 2022 to January 2024). Ethical approval was obtained from the Institutional Ethics Committee (Ref No.: ETHICS/01/2019-20/50). The study was conducted in accordance with STROBE Guidelines.

The following inclusion and exclusion criteria were considered.

Inclusion: Women aged 15-49 years with molar pregnancy confirmed by ultrasonography and histopathological examination, who agreed for follow-up.

Exclusion: Women with incomplete clinical data or having other causes of abortion unrelated to molar pregnancy, including chromosomal abnormalities. 

Eligible participants were consecutively recruited using a non-probability convenience sampling method after written informed consent. Baseline demographics and clinical and laboratory parameters at baseline and follow-up were recorded using a structured proforma that had undergone expert validation. Details of management (suction evacuation, hysterectomy, or chemotherapy) were documented, as well as the complications. Participants were followed with weekly serum β-hCG measurements until normalization (<5 mIU/mL), and histopathology was used to confirm diagnosis and subtype. Cases with persistent disease were classified as gestational trophoblastic neoplasia according to the FIGO (Fédération Internationale de Gynécologie et d’Obstétrique) scoring criteria. Consecutive sampling and standardized diagnostic and follow-up protocols were used to minimize bias. Due to the lack of baseline regional data, all consecutive presentations within the 15-month window were exhaustively recruited.

During clinical examination, uterine size greater than gestational age was defined objectively by a symphysis-fundal height measurement exceeding the calculated amenorrhea weeks by more than 4 cm or via definitive pre-evacuation ultrasonographic volumetry. The requirement for chemotherapy was decided strictly by a diagnosis of gestational trophoblastic neoplasia (GTN) using standard FIGO criteria: i)When the plateau of hCG lasts for four measurements over a period of 3 weeks or longer, that is, days 1, 7, 14, 21; ii)When there is a rise in hCG for three consecutive weekly measurements over a period of at least 2 weeks or more; days 1, 7, 14; iii) If there is a histologic diagnosis of choriocarcinoma.

Statistical Analysis

Data were entered into Microsoft Excel (Microsoft Corporation, Redmond, WA, US) and analyzed using Statistical Package for the Social Sciences (SPSS) version 26 (IBM Corp., Armonk, NY, US). Continuous variables were expressed as mean ± standard deviation (SD) or median (range), depending on distribution, while categorical variables were presented as frequencies and percentages. Comparisons between groups were performed using the independent Student’s t-test for continuous variables and the chi-square test or Fisher’s exact test for categorical variables, as appropriate. The change in serum β-hCG levels before and after evacuation was assessed using the paired t-test. To identify predictors of chemotherapy requirement, univariate logistic regression analysis was initially performed. Variables with p < 0.1 in univariate analysis were included in the multivariate logistic regression model to determine independent predictors. Results were expressed as odds ratios (OR) and adjusted odds ratios (aOR) with 95% confidence intervals (CI). As there was a small number of outcome events, only a few of the clinically relevant variables were included to avoid model overfitting.

The predictive performance of baseline serum β-hCG was evaluated using receiver operating characteristic (ROC) curve analysis, and the area under the curve (AUC) was calculated with 95% confidence intervals. As β-hCG values were recorded in pre-specified categories, midpoint approximation was used to allocate continuous numerical values for ROC analysis. Missing data tracking showed zero attrition for key variables.

Sensitivity, specificity, positive predictive value (PPV), and negative predictive value (NPV) were calculated for the predefined cutoff (≥100,000 mIU/mL), and the optimal cutoff was determined using the Youden index. Agreement between ultrasonography and histopathological diagnosis was assessed using Cohen’s kappa statistic, with values interpreted as poor (<0.20), fair (0.21-0.40), moderate (0.41-0.60), good (0.61-0.80), and very good (>0.80). A p-value of <0.05 was considered statistically significant. As a non-probability convenience sampling method was utilized, the reported 95% Confidence Intervals (CIs) were intended to indicate internal sample precision and data variability rather than serving as statistical inferences for generalizable population parameters. Patients or members of the public were not involved in the design, conduct, reporting, or dissemination plans of the research.

## Results

Out of all screened patients over the 15-month period, 54 met the selection criteria and were recruited for our study. The mean age of the participants was 27.83 ± 5.46 years (range 19-37 years), with 31% in the 21-25 years age group. The majority belonged to the upper-middle socioeconomic class (55.5%) of Kuppuswamy’s scale. Second-gravida constituted the largest group (40.7%), and 66.6% had a prior term pregnancy. Most participants (63%) had no prior history of contraceptive use, though among users, oral contraceptive pills were the most preferred. Baseline demographic characteristics are summarized in Table 1.

**Table 1:** Demographics and clinical features

Variable		N (%)
Age (years) (Mean ± SD)		27.83 ± 5.46
Socio-economic Status (Kuppuswamy Class)	Lower	3(5.6)
	Upper Lower	10(18.5)
	Lower Middle	11(20.3)
	Upper Middle	30(55.5)
Antecedent Pregnancy	Term Pregnancy	36(66.6)
	Non-molar Abortion	7(12.9)
	History of Molar Pregnancy	1(1.8)
Gestational Age at Diagnosis (weeks)	≤8	5(9.3)
	9–12	15(27.8)
	13–16	18(33.3)
	17–20	12(22.2)
	21–24	4(7.4)
	>24	0
Gestational Size vs Uterine Size	Corresponding to gestational age	18(33.3)
	Larger than gestational age	20(37)
	Smaller than gestational age	16(29.6)
Clinical Features	Amenorrhea	54(100)
	Bleeding PV	54(100)
	Hyperemesis	16(29.6)
	Palpitation	6(11.1)
	Passage of Grape-Like Vesicles	16(29.6)
	Abdominal Pain	13(24)
	Pallor	31(57.4)

The mean gestational age at diagnosis was 14.25 ± 3.91 weeks, with most cases diagnosed between 13 and 16 weeks (33.3%). Uterine size was larger than gestational age in 37%. All patients presented with amenorrhoea, followed by vaginal bleeding episodes. Other clinical features included pallor (57.4%), hyperemesis (29.6%), and passage of grape-like vesicles (29.6%).

Baseline serum β-hCG levels showed a wide variation, with a mean of 84,500.40 ± 87,708.03 mIU/mL. Most patients (38.9%) had β-hCG levels between 10,000-100,000 mIU/mL, while 26% had levels ≥100,000 mIU/mL. On ultrasonography, the classic “snowstorm” appearance was observed in 83.3% of cases, and theca lutein cysts were present in 27.5%.

Histopathological examination revealed a complete mole in 75.9% of cases, while choriocarcinoma was detected in 5.5%. Suction and evacuation was the primary treatment modality in 83.3% of patients, whereas 16.7% had a total abdominal hysterectomy. Chemotherapy was required in 22.2% of patients, of which 41.6% required multi-agent regimens based on FIGO risk stratification. Crucially, all choriocarcinoma cases were confirmed via primary histopathology of the tissue immediately following initial uterine evacuation, rather than developing during secondary follow-up. Treatment characteristics are tabulated in Table 2.

**Table 2:** Treatment characteristics β-hCG: β-human chorionic gonadotropin

Parameter	Characteristic	N (%)
Pre-evacuation β-hCG (IU/L)	<1000	4 (7.4)
	1000-10,000	15 (27.8)
	10,000-100,000	21 (38.9)
	>100,000	14 (26)
Ultrasonography & Chest X-ray Findings	Theca lutein cyst	15 (27.5)
	Snowstorm appearance	45 (83.3)
	Invasive mole	1 (1.8)
	Metastatic lesions in the lung	0
Treatment Given	No further intervention (only suction & evacuation)	45 (83.3)
	Total abdominal hysterectomy (TAH)	9 (16.7)
	Chemotherapy	12 (22.2)
Chemotherapy Regimen	Single agent	7 (58.3)
	Multi-agent	5 (41.6)

Meticulous patient tracking resulted in zero loss-to-follow-up. All 54 enrolled patients fully completed their scheduled post-evacuation β-hCG surveillance until absolute normalization or till clinical progression to values necessitating chemotherapy.

There was a statistically significant reduction in β-hCG levels following evacuation, with a mean decline of 51,210.68 ± 58,945.24 IU/L at 48 hours (95% CI: 35,121.69-67,299.60; p < 0.001). The mean time to normalization of β-hCG was 11.55 ± 3.41 weeks, with 48.1% achieving it within 9-12 weeks. Trends in β-hCG decline are presented in Table 3, and treatment comparisons by mole type are presented in Table 4. Comparative analysis between complete and partial moles demonstrated no statistically significant differences in age, gestational age, baseline β-hCG levels, time to normalization, risk classification, or requirement for blood transfusion.

**Table 3:** β-hCG levels before and 48 hrs after evacuation β-hCG: β-human chorionic gonadotropin

Variables	Pre-evacuation	48 hrs post-evacuation
N	54	54
Mean±SD	84500.40+87708.03	33289.75+31866.50
Median	59348.50	23186.50
Range	950 - 2,75,000	275 - 97,000
Mean difference (95% CI)	51210.68+58945.24(35121.69,67299.60)	
P-value (two-sided)	<0.001	

**Table 4:** Comparison of patients with complete and partial molar pregnancy β-hCG: β-human chorionic gonadotropin

Factor	Complete Mole (n=41)	Partial Mole (n=9)	P-value
Age (Years)	28.29+5.48	25.66+4.76	0.190#
Gestational age (Weeks)	14.09+3.84	12.66+4.12	0.323#
Baseline β-hCG (IU/ml)	76101.34+85216.14	94144.22+80892.22	< 0.547#
Time to normalize β-hCG (Weeks)	11.22+3.12	11.67+3.75	< 0.710#
High Risk	3 (75%)	1(25%)	0.704*
Need for Blood Transfusion	19 (46.6%)	4 (44.4%)	0.918*
#- Independent T-test *- Chi-square test			

Univariate analysis showed a serum β-hCG of ≥ 100,000 mIU/mL (OR 5.8, 95% CI 2.1-15.9, p = 0.001), uterine size greater than gestational age (OR 3.2, 95% CI 1.2-8.5, p = 0.02), and presence of theca lutein cysts (OR 2.9, 95% CI 1.1-7.6, p = 0.03), which were significantly associated with chemotherapy requirements. On multivariate regression, serum β-hCG ≥100,000 mIU/mL was the strongest independent predictor (adjusted OR 4.9, p = 0.003), followed by uterine size greater than gestational age (adjusted OR 2.8, p = 0.048). The association with theca lutein cysts was not statistically significant (p = 0.08). This has been tabulated in Table 5. No patients were lost to follow-up due to the strict follow-up protocols adhered to.

**Table 5:** Multivariate logistic regression analysis β-hCG: β-human chorionic gonadotropin

Variable	aOR	95%CI	p-value
β-hCG ≥100,000	4.9	1.7-13.8	0.003
Uterine size > GA	2.8	1-7.9	0.048
Theca lutein cyst	2.3	0.9-6.1	0.08

**Figure 1:**
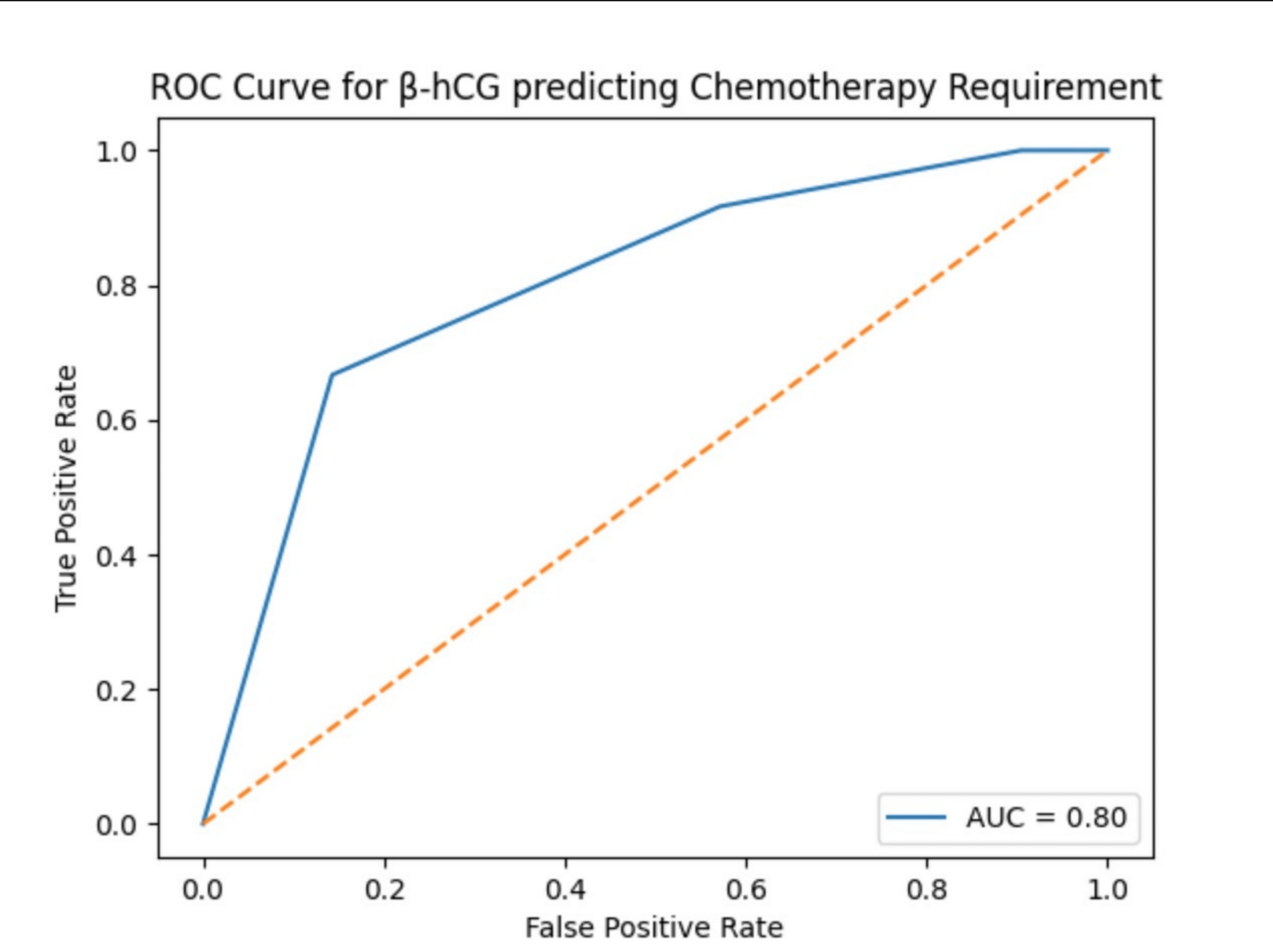
ROC curve ROC: receiver operating characteristic

The ROC curve (Figure 1) demonstrated an AUC of 0.78 (95% CI: 0.65-0.91, p < 0.001), indicating good discriminatory ability. As β-hCG values were recorded in categories, midpoint approximation was used for analysis. Sensitivity and specificity were 66.7% and 81%, respectively, for the predefined cutoff of β-hCG ≥100,000 mIU/mL. The positive and negative predictive value was 53.8% and 88.6%. The Youden index deduced optimal cutoff was approximately 75,000 mIU/mL, which had a sensitivity of 75% and specificity of 74%. A kappa value of 0.62 (p < 0.001) suggested that ultrasonography is a reliable initial diagnostic modality.

## Discussion

Our study reports a hospital-based prevalence of molar pregnancy of 10.71 per 1,000 live births, which is substantially higher than that documented in previous studies [9-11]. This disparity likely reflects an institution-specific burden rather than true population incidence, and must be interpreted cautiously as our center's referral capability naturally overestimates the broader population-level burden. Heightened clinical suspicion, coupled with the increasing availability of high-resolution imaging, may have contributed to higher case detection.

The age distribution in this study, with a peak in the 21-25 years group, is consistent with some studies [12,13] but contrasts with others [14] who reported increased risk at advanced maternal age. This discrepancy highlights the possibly complex relationship between maternal age and molar pregnancy, suggesting that this could be influenced by regional, nutritional, or genetic factors [14,15]. Extremes of reproductive age as one of the principal risk factors have been reported.

The socioeconomic profile observed in our population had a predominance of upper-middle-class patients from the conventional association of molar pregnancy with lower socioeconomic status [16]. This may be explained by increasing access to healthcare, where women from higher socioeconomic strata are more likely to seek care at tertiary centers and may undergo early diagnostic evaluation. The socioeconomic gradients observed in tertiary hospital-based studies like ours may stress patterns of healthcare utilization rather than true disease distribution.

Reproductive characteristics in our subjects further show the heterogeneity of risk factors. The predominance of multigravid women contrasts with earlier reports [15], suggesting a higher incidence in primiparous women, indicating that parity alone may not be a reliable determinant of risk. Instead, molar pregnancy likely arises from a complex interplay of genetic and environmental factors [9,11,16]. The low recurrence rate observed aligns with existing literature, reinforcing that while prior molar pregnancy is a known risk factor, its overall contribution at the population level remains limited [13].

Clinically, the presence of amenorrhoea and vaginal bleeding reasserts these as cardinal features of molar pregnancy, even with the advent of advanced imaging. The persistence of classic symptoms, such as uterine size greater than gestational age and passage of vesicles in a substantial proportion of patients, suggests that despite advances in diagnostic modalities, many cases continue to present after symptomatic progression [14]. This highlights potential gaps in early antenatal surveillance, particularly in resource-constrained or geographically remote settings [17].

The biochemical and radiological findings in this study are in accordance with reports [14,18]. Markedly elevated β-hCG levels and the high prevalence of the classical “snowstorm” appearance confirm the usefulness of these markers. The predominance of complete mole is clinically significant, given its higher malignant potential, and underscores the importance of histopathological confirmation in guiding prognosis and follow-up [19].

Management outcomes in this cohort are in accordance with standard care protocols [19,20], with suction and evacuation as the primary intervention. The proportion of patients requiring chemotherapy is comparable to existing literature [21], and the predominance of low-risk cases according to WHO scoring indicates favourable outcomes when appropriate treatment pathways are followed. The significant early decline in β-hCG levels further supports the effectiveness of prompt uterine evacuation. Importantly, our follow-up data demonstrate that most patients achieved normalization of β-hCG within 9-12 weeks, consistent with existing findings [14]. This emphasises the important role of structured surveillance in preventing progression to gestational trophoblastic neoplasia [21]. The absence of significant differences between complete and partial moles in terms of β-hCG levels, as recorded before [18], suggests that close follow-up remains essential regardless of histological subtype. While our comparative analysis showed no significant variations between complete and partial moles across baseline markers, these subgroup interpretations are limited by the small size of the partial mole cohort, which inherently reduces the statistical power to detect subtle clinical differences. In the present study, serum β-hCG ≥100,000 mIU/mL was the strongest predictor of chemotherapy requirement, both on univariate and multivariate analysis, pointing to its role as an indicator of trophoblastic proliferation and tumor burden. The ROC curve analysis further showed its importance as a predictive biomarker, as in reports [20]. Uterine size greater than gestational age also independently predicted the need for chemotherapy, signifying increased disease volume in our population. The observed chemotherapy initiation rate is at the higher end of reported literature. This could be attributed to referral bias; as a premier tertiary teaching facility in a remote, geographically isolated terrain, our department receives a disproportionate volume of advanced, highly symptomatic, or high-risk presentations where early primary care intervention was unavailable.

Furthermore, the moderate agreement observed between ultrasonography and histopathology highlights that while ultrasound remains a reliable and practical initial diagnostic tool, histopathological confirmation continues to be essential for definitive diagnosis.

Strengths and limitations

This study adds valuable prospective data from a geographically underrepresented region, contributing to the limited evidence base on molar pregnancy in Northeast India and resource-limited settings elsewhere. The systematic data collection and follow-up strengthen the reliability of findings. However, our study has important limitations. It is a single-center design with a small overall sample size. Given that only 12 primary clinical events required chemotherapy intervention, our multivariate logistic regression model faces an inherent risk of overfitting; hence, these predictive coefficients should be interpreted as exploratory rather than definitively causal. Furthermore, pre-evacuation serum β-hCG values recordings in broad tiers required the use of midpoint approximations in our ROC analysis, which limits the analytical precision of our predictive curves. Finally, significant selection and referral biases arising from our tertiary-center status mean these outcomes reflect specialized institutional burden rather than community-wide regional data. Though no missing data were observed for key variables in our study, residual bias due to categorized β-hCG values may have been present.

Implications for practice and research

The findings highlight the importance of strengthening early antenatal screening and improving access to imaging, particularly in peripheral and rural areas in primary care settings. Given the potential for progression to gestational trophoblastic neoplasia, establishing robust follow-up systems for β-hCG monitoring is essential. Future research should focus on multicentric studies with larger sample sizes to better delineate risk factors and validate predictive models for disease progression.

## Conclusions

Molar pregnancy in this setting predominantly affects younger women and presents with classical clinical and biochemical features. Early diagnosis and appropriate management result in favorable outcomes; however, the relatively high prevalence observed underscores the influence of referral patterns and healthcare access. Strengthening early detection and ensuring adherence to follow-up protocols remain critical to reducing morbidity associated with this condition.
